# Total Knee Replacement as Treatment for Hemophilic Arthropathy: A Case Series

**DOI:** 10.7759/cureus.72332

**Published:** 2024-10-24

**Authors:** Murali Govindasamy, Kunalan Ganthel Annamalai, Lynn Azura Binti Md Sham

**Affiliations:** 1 Department of Orthopedics, Hospital Kuala Lumpur, Kuala Lumpur, MYS; 2 Department of Orthopedics, Sarawak General Hospital, Kuching, MYS; 3 Department of Orthopedics, Ampang Hospital, Kuala Lumpur, MYS

**Keywords:** hemophilic arthropathy, joint replacement, knee society score, short-term results, total knee replacement

## Abstract

Background and objectives

Hemophilic arthropathy is a known complication of patients with hemophilia, with the knee as the commonest joint affected. Patients with this condition have severe pain and restricted joint mobility, affecting their activities of daily living. Total knee replacement can increase these patients’ quality of life. Nevertheless, this surgery has a high risk of complications due to the nature of the underlying hemophilia. In this study, we present the short-term outcome of a case series of six patients with hemophilic arthropathy who underwent total knee replacement at a hospital with hematology support performed by an experienced arthroplasty surgeon.

Patients and method

This study reviewed and included eight consecutive total knee replacements in six patients with hemophilic arthropathy performed by a single board-certified arthroplasty surgeon from 2019 to 2023. The demographic profile of the patients, pre- and postoperative range of movement, and short-term outcome scores were summarized.

Results

The mean preoperative flexion contracture was 10.6° (range: 5°-30°), and it was 0.7° (range: 0°-5°) postoperatively. The mean preoperative flexion of the knee was 78.1° (range: 45°-110°) and 98.8° postoperatively. At six-month follow-up, the average Knee Society Score expectation, satisfaction, and functional scores had improved from 8.3 to 13.8 points, 20 to 38.5 points, and 42.8 to 80.5 points, respectively.

Conclusion

This study suggests that short-term results of total knee replacement in patients with hemophilic arthropathy are favorable, with increased range of movement and quality of life. We also conclude that surgery should be performed by an experienced surgeon with the support of a dedicated hematology unit.

## Introduction

Hemophilia A and B are rare X-linked inherited bleeding disorders caused by complete or partial deficiency in or the absence of coagulation factors VIII and IX. The improvement of medical management, mainly the advent of recombinant factors, has significantly improved the quality of life and reduced complications for hemophilic patients. These recent advancements have also moved the management of hemophilic patients from life-saving to joint preservation. Nevertheless, despite these advancements, patients with severe hemophilia (factor <1U Dl) still present with recurrent hemarthrosis and its sequelae of joint destruction [1]. Although it can affect various joints, the knee joint is the most commonly affected joint, accounting for almost 80% of hemophilic arthropathy [2]. The pathophysiology of this condition is such that the synovial insult causes repeated joint bleeding resulting in synovial hypertrophy. With every bleed, the joint is predisposed to joint destruction and arthropathy. In other words, the mechanism of this injury is an unrelenting vicious cycle [1].

Patients with hemophilic arthropathy are those of younger age with a higher demand, as many are still employed and are keen to engage in physical activities. They also present with severe pain and restricted range of movement, and this affects their activities of daily living [3,4]. This presents a unique problem that needs to be addressed to improve patients’ function. The joint-preserving surgeries for hemophilic arthropathy include synovectomy, radiosynovectomy, and contracture release, but these procedures are temporary and do not significantly improve patients’ quality of life. The only option available in end-stage hemophilic arthropathy is to perform joint replacement surgery [4]. Literature has shown that total knee replacement in patients with hemophilic knee arthropathy is effective in reducing pain and improving function [3-8].

Due to the unique nature of hemophilia, surgery in this group of patients carries a high risk of complications such as infection, life-threatening bleeds, and implant failure [4,5,8,9]. To mitigate the high risk of perioperative complications of surgery, the current recommendation by the World Hemophilia Federation is that joint replacements should only be performed in recognized hemophilia treatment centers [10]. This is because the patient will require a specialized laboratory for close monitoring of factor levels as well as readily available factors for aggressive transfusion. Patients with hemophilic arthropathy also frequently present with deformities in multiple planes. The typical deformity is flexion contracture and external rotation of the tibia. Joint surface destruction can be severe, causing cysts and bone loss leading to dorsal subluxation of the tibia. Therefore, total knee replacement surgery in patients with hemophilic arthropathy should only be performed by an experienced surgeon, as it requires advanced surgical skills.

At Kuala Lumpur Hospital, there is no dedicated hematology unit to manage hemophilic patients. The nearest regional center for hematology is Ampang Hospital, where patients with hemophilia are managed. Hemophilic patients who require total knee replacement are co-managed by the Joint Replacement Unit of Kuala Lumpur Hospital, the Orthopedic Department of Ampang Hospital, and the Hematology Unit of Ampang Hospital. This collaboration is to ensure that these complex cases yield the best outcome while reducing the risk of complications. We would like to present a case series of eight total knee replacements in hemophilic arthropathy patients that were performed at Ampang Hospital to demonstrate improvement in range of movement and Knee Society Score (KSS).

## Materials and methods

Patients

All consecutive patients with hemophilic arthropathy who underwent total knee replacements at Ampang Hospital by a board-certified arthroplasty surgeon with 12 years of experience were included in this study. Informed consents were obtained from all patients for the publication of this article. All patients were diagnosed with severe hemophilia based on the World Federation of Hemophilia guidelines as per Table 1 [10].

**Table 1:** Classification of hemophilia severity and bleeding risks

Severity	Clotting factor level	Bleeding episodes
Severe	<1 IU/dl (<0.01 IU/mL) or <1 % of normal	Spontaneous bleeding into joints or muscles, predominantly in the absence of identifiable hemostasis challenge
Moderate	1-5 IU/dl (0.01-0.05 IU/mL) or 1-5 % of normal	Occasional spontaneous bleeding; prolonged bleeding with minor trauma or surgery
Mild	5-40 IU/dl (0.05-0.40 IU/ml) or 5-40 % of normal	Severe bleeding with major trauma or surgery; rare spontaneous bleeding

Hematological management

After stringent preoperative evaluation by the hematology team, factor VIII was transfused to achieve the level of 100%. Intraoperatively, factor levels were monitored closely by a hematologist, with intraoperative transfusion carried out if factor levels dropped below 60%. Postoperatively, the patient was transferred to the hematology ward and monitored for 14 days. During this time, factor levels were monitored and maintained above 60%. A drop in hemoglobin level was managed with a quick transfusion.

Surgical procedure

Surgery was carried out using the standard midline incision with total synovectomy done as part of the approach. For all cases, posterior sacrificing implants were used. All implants were cemented using antibiotic-loaded cement and prophylactic intravenous antibiotics (first-generation cephalosporin) were prescribed upon induction and continued for the first 24 hours. The operation was carried out under tourniquet control and no anti-thrombotic chemoprophylaxis was given postoperatively. A surgical drain was placed on closure and removed within 24 hours of surgery. Patients requiring intervention on both knees were done as staged procedures with a minimum of six-month gap between surgeries.

All patients were started on guided active exercises at day one post-surgery, and this was maintained for at least six weeks post-surgery. Patients were allowed partial weight bearing using a walking frame from day one post-surgery. They were then gradually allowed to progress to full weight bearing as tolerated within six weeks.

Data collection

All patients were followed up for at least six months after the surgical intervention. The demographic data, preoperative range of movement, and implant types were obtained from the medical records. Patient satisfaction, expectation, and functional scores were determined using the 2011 Knee Society Score preoperatively and six months postoperatively. Range of movement was also measured at this point, and patients were continuously monitored for any complications. There were no patients lost to follow-up.

## Results

A total of six patients were enrolled in this study, with ages ranging from 24 to 41 years at the time of surgery. All patients had severe hemophilia A and were on twice-weekly self-administered factor VIII infusion therapy. Fortunately, none of the patients developed antibodies toward factor VIII, nor did they contract any blood-borne infections. Four patients had only one knee operated on, while two patients had bilateral total knee replacements done at least six months apart. Only one patient required a constrained liner bilaterally, while all other patients had a posteriorly stabilized knee. Table 2 summarizes patients’ demographics (age and gender), comorbidities, side of surgery, and type of implant used.

**Table 2:** Patients demographic, comorbidity, and side of surgery and type of implant used

Patient	Age	Gender	Side of surgery	Comorbidities	Implant
1	41	Male	Bilateral	Hypertension	Constrained bilaterally
2	29	Male	Left	Nil	Posterior stabilized
3	34	Male	Bilateral	Asthma	Posterior stabilized bilaterally
4	27	Male	Right	Nil	Posterior stabilized
5	24	Male	Right	Nil	Posterior stabilized
6	31	Male	Left	Asthma	Posterior stabilized

Table 3 summarizes the three components of the 2011 Knee Society Score. At six-month follow-up, the average KSS expectation, satisfaction, and functional scores had improved from 8.3 to 13.8 points, 20 to 38.5 points, and 42.8 to 80.5 points, respectively. One patient with bilateral total knee replacement had a maximal satisfaction score. All patients had an increased functional score postoperatively, although none achieved a full functional score. Both patients who underwent their second knee replacement did not have any improvement in their postoperative expectation scores.

**Table 3:** Preoperative and postoperative KSSS, KSES, and KSFS KSSS: Knee Society Satisfaction Score, KSES: Knee Society Expectation Score, KSFS: Knee Society Functional Score

Patient	Side of surgery	Preoperative KSES	Postoperative KSES	Preoperative KSSS	Postoperative KSSS	Preoperative KSFS	Postoperative KSFS
1	Left	5	14	14	38	37	75.5
1	Right	14	14	14	38	37	75.5
2	Left	7	13	26	38	52	80
3	Right	7	14	28	40	42	88
3	Left	14	14	28	40	42	88
4	Right	6	13	16	38	42	87
5	Right	5	13	16	38	44	72
6	Left	8	15	18	38	46	78

Postoperatively, none of the patients developed hemarthrosis requiring surgical intervention. Two patients required a single pint of intraoperative packed cell transfusion, and one patient required 2 pints of packed cell transfusion postoperatively. At the time of writing, no patient developed an infection or implant failure or required any revision.

## Discussion

Hemophilic arthropathy is a complex condition that is the end sequelae of a unique chronic pathophysiological process [10]. Due to the nature of the disease, multiple repeated intra-articular bleedings occur, which results in hemosiderin and iron deposition in joints. This in turn leads to upregulation of pro-inflammatory cytokines, leading to synovial hypertrophy and articular cartilage destruction. As the disease progresses, the patient develops severe pain as well as limited range of movement, thus reducing mobility and quality of life [1,3,7,9]. This is also demonstrated in our case series, where patients had very low satisfaction and functional scores preoperatively.

Patients with hemophilic arthropathy are usually males of a younger age group, and this is demonstrated in our series of patients who had a median age of 31. This is similar to Rodriguez-Merchan et al., in a series of 35 knees where patients had a mean age of 31 [11]. Due to their young age, hemophilic arthropathy patients have a higher functional demand, require higher mobility, and are independent and more physically active. Joint-preserving surgeries are temporary measures and do not increase the quality of life significantly [3,4]. Total knee replacement is the only surgical intervention proven to give patients significant benefits. Goddard et al. followed up patients for up to 25 years and showed good to excellent Hospital for Special Surgery scores in 95% of the patients [2]. Our case series follows this positive pattern, as all patients have increased satisfaction scores. Patients also have a better quality of life, signified by functional score improvement.

Despite good outcomes, total knee replacement in hemophilic patients carries a greater risk of complications as compared to the general population, such as hemarthrosis, fractures, infection, and malalignment. Moore et al., in a meta-analysis of 336 knees, showed a complication rate of 31% [9]. The rate of prosthetic joint infection after primary total knee replacement in hemophilic patients is about 11% compared to 1% in non-hemophilic patients [6]. The incidence of infection is also closely linked to the occurrence of hemarthrosis [2,6]. Thus, it is essential to have close monitoring perioperatively by a hematology team in order to prevent hemarthrosis and infection. In our case series, an aggressive infusion of factor VIII was done by the hematology team to ensure factor levels remained above 60%.

Currently, with the advent of newer implants as well as improvements in surgical techniques, complication rates have significantly reduced. Fenelon et al. showed in a meta-analysis that operative interventions done after the year 2000 had a much lower complication rate than those done prior to the year 2000 [1]. In our series, all cases were done via the cruciate sacrificing method with at least a posterior stabilized implant. We also prepared constrained, non-hinged implants, stems, and augments. These additional implants may be required if the joint destruction causes severe bone defects or the surgical step requires extensive soft tissue release to balance the contracted knee. As the surgical technique can be quite challenging, the surgery should be performed by a surgeon with vast experience in knee arthroplasty to reduce the risk of complications [12].

The best predictor of patients’ postoperative range of movement is their preoperative range of movement [13]. Hemophilic arthropathic patients tend to have a lower range of movement postoperatively when compared to non-hemophilic patients undergoing total knee replacement. This is due to severe muscular atrophy and flexion contracture in these patients caused by the numerous intra-articular bleeding episodes [6,14]. This trend follows in our study, where patients with a lower postoperative range of movement also had a low range of movement preoperatively. Nevertheless, all patients still demonstrated improvement in their range of movement post-surgery. Kubes et al. also specifically demonstrated improvement in the range of movement in short-term follow-up of patients with hemophilic arthropathy who underwent total knee replacement [8].

The limitations of our case series include a small sample size and being a retrospective study. Due to the rarity of this condition, it may be difficult to conduct a large study in a single center and may require a nationwide multi-center prospective study to ensure a better statistical analysis.

## Conclusions

From our case series, we can conclude that total knee replacement is a good option for patients with hemophilic arthropathy. This procedure can improve the patient’s range of movement, function, and quality of life. To achieve a positive outcome and lower the risk of complications, the surgery should be performed by an experienced joint replacement surgeon at a dedicated hematology center.
